# A Multi-Module Fixed Inclinometer for Continuous Monitoring of Landslides: Design, Development, and Laboratory Testing

**DOI:** 10.3390/s20113318

**Published:** 2020-06-10

**Authors:** Giuseppe Ruzza, Luigi Guerriero, Paola Revellino, Francesco M. Guadagno

**Affiliations:** 1 Department of Sciences and Technologies, University of Sannio, 82100 Benevento, Italy; paola.revellino@unisannio.it (P.R.); guadagno@unisannio.it (F.M.G.); 2 Department of Earth, Environment and Resources Sciences, University of Naples, Federico II, 80126 Naples, Italy; luigi.guerriero2@unina.it

**Keywords:** inclinometer, micro-electromechanical systems (MEMS), Arduino^®^, inclination monitoring, landslides, continuous monitoring

## Abstract

Continuous monitoring of landslides is of basic importance for understanding their behavior, defining their 3D geometry, and providing a basis for early warning purposes. While a number of instrumentations can be used for tracking surface displacement, only automatic or fixed multi-module inclinometers can be used for continuous monitoring of displacement at depth, providing valuable information for landslide geometry reconstruction. Since these instruments are very expensive, thus rarely used, a low-cost and multi-module fixed inclinometer for continuous landslide monitoring has been developed. In this paper, the electronics of the system, including sensor characteristics and optimization, controlling software, and structure are presented. For system development, a single module prototype was first developed and tested in the field to ensure sufficient measuring performance. Subsequently, the multi-module system was designed, assembled, and tested in controlled conditions. Test results indicate the good performance of the system with a displacement measuring accuracy of 0.37% of the length of the inclinometer chain. The linearity test indicates the high linearity of the measures, especially in the range ±20°, which is the typical operating range of such kinds of instrumentations. The thermal efficiency test indicates the high efficiency of the system in preventing measuring errors caused by thermal drifting.

## 1. Introduction

Landslides exhibit highly variable velocities [1]. Some landslides persistently move at a low rate for long periods, while others exhibit short-lived movement with velocities up to several meters per second [2,3]. In particular conditions, landslides can move in a slow persistent manner for a few to several years with only minor short-lived accelerations. Alternatively, slow movement can culminate in a catastrophic failure [4]. The spatial and temporal variability of the rate of movement depicts the kinematics of the event. It is closely related to landslide damage potential, and to accurately describe it, at least several years of continuous monitoring data are required [5,6]. In absence of monitoring data, spatial distribution of the movement can be inferred by landslide surface mapping [7,8]. Since an accurate analysis of landslide-related risk should account for the magnitude of the events, factors controlling movement, and potential near-future evolution, a detailed knowledge of landslide kinematics and dynamics through continuous monitoring is of basic importance and can contribute to mitigation measures design [9]. Despite its importance, landslide displacement monitoring is not always applied due to its expense and the need for integration with additional sensors to monitor factors controlling movement. On this basis, a number of authors have explored the potential of open-source platforms-based, low-cost monitoring systems that, after appropriate treatment (e.g., calibration), can be used in landslide displacement monitoring applications [10].

Landslide displacement monitoring can be accomplished with a number of instrumentations (GPS, Robotic Total Positioning Station (rTPS), extensometers, etc.; e.g., [11,12]). However, most of them (i) do not allow nearly continuous monitoring; (ii) imply time-consuming and expensive monitoring campaigns; and/or (iii) cannot be integrated with additional sensors [9]. Wire extensometers are often used in landslide monitoring since they allow continuous displacement recording. These sensors, associated with specific datalogging units, can be used when landslide displacement is concentrated along a well-defined shear surface and can be integrated with additional sensors (e.g., pore pressure transducer) for landslide dynamic analysis [13]. However, they acquire data at the landslide surface, making it impossible to decipher displacement distribution at depth and to identify and localize a single or multiple sliding surfaces. In addition, except for a low-cost system proposed by the authors of [10], the wire extensometer has the disadvantage of being very expensive with a cost of around €1000 for a single monitoring point. To overcome the problem of deciphering displacement distribution at depth and identifying the sliding surface, traditional inclinometers can be a valid alternative [14]. However, with traditional inclinometers, only discrete measurements can be achieved with a standard, human-operated single module probe, and automatic measuring units or multi-module near-real-time operating systems can be very expensive and of complex use [15].

Multi-module systems are typically composed of multiple biaxial tilt measuring units that, forming a chain, can be installed within a borehole equipped with a specific inclinometer housing. Once installed, the system continuously acquires tilt data at different depths and converts them into a displacement. Cumulating displacement data along the chain, the total displacement at the surface can be derived [16]. These systems have been used in a number of landslide monitoring projects proving their potential to provide information about the number and location of sliding surface [17,18]. The use of such kinds of systems for early warning purposes is less common, even though results from the authors of [19], who developed a shallow monitoring system based on tilt sensors, indicate that these tools are promising also in landslide monitoring for early warning purposes.

In this paper, starting from precedent works of the authors of [20,21], who analyzed micro-electromechanical system (MEMS) accelerometer behavior in an inclination measurement perspective, providing a strategy of thermal drift reduction that makes them suitable for field monitoring, we present a newly developed, low-cost, multi-module inclinometer, suitable for continuous landslide monitoring. This system can support landslide process understanding, including basal slip surface identification and movement tracking, and can be easily implemented with a real-time data transfer module for early warning purpose. The relevant and novel aspects of the developed system are related to its low-cost and open-source nature that allow future improvements of both software and hardware features and makes the system adaptable to specific monitoring applications and different site conditions. For its development, a specific MEMS Inertial Measurement Unit (IMU) sensor board was selected, and a single module monitoring prototype, based on the selected components, was assembled and tested in the field. After evaluating the single-module prototype test results, through comparison with a commercial sensor, the multi-module inclinometer was assembled and tested in controlled conditions.

## 2. Single Module Prototype

### 2.1. Hardware and Software

**Figure 1:**
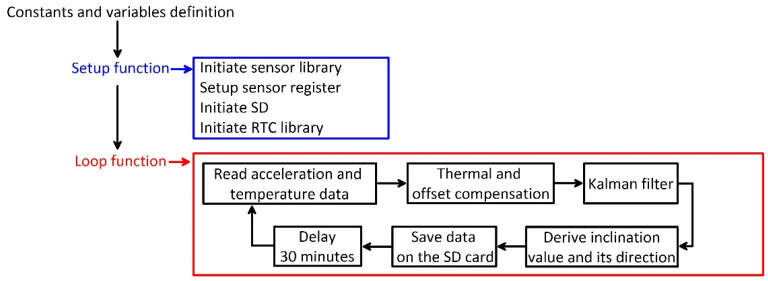
Flow chart showing the logic of the code of the single module prototype.

**Figure 2:**
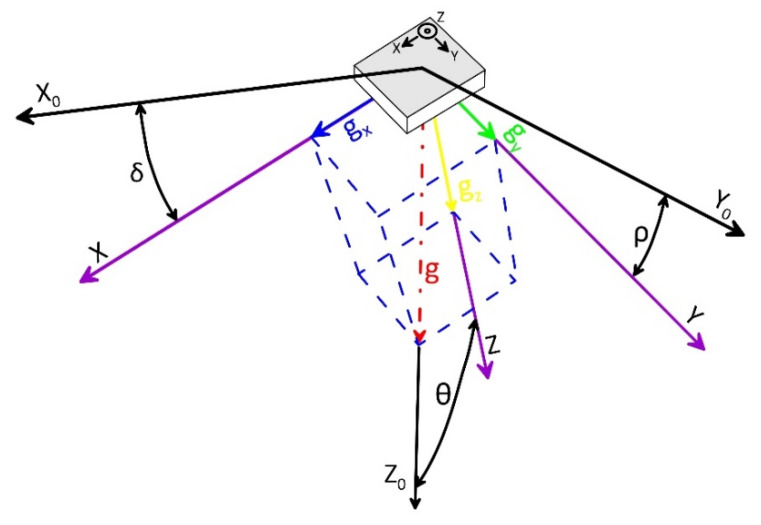
Standards for onboard micro-electromechanical system (MEMS) accelerometer readings and inclination calculation. The blue, green, and yellow vectors are the components of the Earth’s gravity (red dash–dot arrow) measured by each axial accelerometer.

The single module prototype is composed of (i) a MEMS sensor; (ii) an Arduino^®^ board; (iii) a local storage shield with a selectable memory capacity; and (iv) a power system. The measuring and logging electronic components are housed within a 10 cm × 10 cm × 7 cm waterproof box, and connection to the power system, housed within a separated, larger, waterproof box, is guaranteed by a waterp cabling system. The sensor selected for inclination monitoring, a LSM9DS0 MEMS IMU (STMicroelectronics, Geneva, Switzerland) [22], was soldered on a compatible pin hole Arduino^®^ proto shield, installed on the Arduino^®^ board for integration purposes. The power system is a stand-alone electrical source composed of a 50 W solar panel and a 12 V, 12 A lead battery. Battery recharge is managed by a 5 A max regulator. The selected sensor, an LSM9DS0 MEMS IMU has been already tested by the authors of [20] for understanding capabilities of onboard accelerometers in inclination measurement. The good performance after thermal optimization obtained by the authors of [21] indicated the suitability of this MEMS IMU in a field monitoring perspective. This IMU has nine degrees of freedom and is equipped with three accelerometers, three gyroscopes, three magnetometers, and a temperature sensor. It provides a digital output through the I2C communication protocol and has an onboard 16-bit Analog to Digital Converter (ADC). The maximum range of onboard accelerometers could be set at ±2, ±4, ±6, ±8, or ±16 g. This IMU sensor was selected because of the quite high resolution of the ADC, that allows a resolution of 0.061 g/least significant bit (LSB) in the setting range ±2 g. For system development, an Arduino^®^ Uno board was used as reading and data processing interface [23]. This board has been chosen because of its simple use and low cost. The logic of the code developed for making the prototype work is reported in Figure 1. The code was developed and compiled using the Arduino^®^ IDE environment. After first initialization of the variables, the libraries, acceleration, and temperature data are acquired by the sensors and read through the Arduino^®^ board. Such data are optimized for thermal drifting and corrected for the presence of a measuring offset before being used for estimating inclination angles (*δ*, *ρ*, and *θ*; Figure 2). Such angles are used for estimating the inclination and its direction in space.

### 2.2. Sensor Optimization and Inclination Estimation

As indicated by the authors of [21], the selected MEMS IMU sensor board needs to be optimized in order to be used in monitoring applications both in terms of temperature compensation and offset correction. On this basis, the sensor board has been analyzed for deriving specific compensation coefficients in a thermal drifting mitigation perspective following the strategy proposed by the authors of [20]. Especially, the analyses of the MEMS IMU sensor board were completed using a specifically developed automatic thermal chamber [21]. The chamber allowed to test the thermal behavior of onboard accelerometers in a defined temperature range and variating sensor position in space during test. This is of particular significance for studying the behavior of multi-axial MEMS IMU onboard accelerometers including potential correlation between inclination and thermal drifting [21]. In order to obtain thermal calibration coefficients, acquired data were fitted by a low degree polynomial equation [20]. Equation parameterization was automatically competed using the Curve Fitting Toolbox implemented in Matlab™. The onboard sensors were calibrated in a temperature range between −10 to 45 °C. This range has been chosen in order to be representative of field measuring conditions in most of underground and subaerial applications. According to observations of the authors of [20], after thermal compensation, Root Mean Square Error (RMSE) in estimating inclination along each single axial accelerometer should decrease; for this sensor typology, around 90%.

After optimization, acceleration data were used for estimating inclination of the MEMS IMU in space following standards of Figure 2. For converting acceleration data into inclination angles, multiple algorithms are available. These algorithms are based on the inverse tangent function, on the inverse sine function, or inverse cosine function [24]. Since the used sensor, equipped with three accelerometers, provides acceleration value along three orthogonal axes (i.e., *x*, *y*, and *z)*, the angular values *δ*, *ρ*, and *θ* can be calculated with equations based on inverse tangent function (i.e., algorithms based on triple-axis method [25]). This algorithm has been chosen because of the accuracy and stability of the obtained results, the low computation resources needed in comparison with other algorithms, and its constant effective incremental sensitivity [26,27]. The equations below (1, 2, and 3) have been used to convert the gravity component on the axes in angular values:

(1) $$\delta =tan^{-1}\left(\frac{A_{x}}{\sqrt{A_{y}^{2}+A_{z}^{2}}}\right)$$

(2) $$\rho =tan^{-1}\left(\frac{A_{y}}{\sqrt{A_{x}^{2}+A_{z}^{2}}}\right)$$

(3) $$\theta =tan^{-1}\left(\frac{\sqrt{A_{y}^{2}+A_{x}^{2}}}{A_{z}}\right)$$

In these equations, $A_{x}$, $A_{y},$ and $A_{z}$ are gravity components measured by each axial accelerometer; δ is the angle between the *x* axis of accelerometer and the horizontal line; ρ is the angle between the horizontal line and the *y* axis; and θ is the angle between the *z* axis and gravity vector of the Earth (Figure 2). The estimation of the three position components of the sensor (i.e., acceleration measured by each single axial accelerometer) allows the estimation of the magnitude of the inclination and its direction in space.

Since performance of MEMS accelerometers can be influenced by potential offset that generally affects a single or multiple axial sensors, after thermal compensation, offset analysis and correction was completed. Equations (1)–(3) have been used for estimating inclinations in the assumption that the resultant of Earth’s gravitational acceleration components, measured by each single axial accelerometer, equal 1 g (≈9.8 m/s^2^). Equation (4) can be used to calculate the resultant acceleration components from the axial measurements in static conditions:

(4) $$A_{g}=\sqrt{A_{x}^{2}+A_{y}^{2}+A_{z}^{2}}=1\;g$$

where $A_{g}$ is the acceleration gravity; $A_{x}$, $A_{y}$, and $A_{z}$ are the acceleration gravity components measured on the *x*, *y*, *z* axes, respectively. On this basis, the magnitude of the bias induced by the offset can be estimated as difference between the value of $A_{g}$ obtained using Equation (4) and the accelerometric data measured in static position. In this case, the 3D ellipsoid fitting method was applied that, minimizing a cost function, provides the real values of the estimation [28,29]. Especially, the real values measured along each single axis was calculated considering the bias to the vector of the non-corrected accelerometer data (*x*, *y*, *z*) multiplied to a transformation matrix containing the offset estimate, as reported below:

(5) $$\left[\begin{array}{c} A_{x}\\ A_{y}\\ A_{z} \end{array}\right]=\left[\begin{array}{ccc} A_{11} & A_{12} & A_{13}\\ A_{21} & A_{22} & A_{23}\\ A_{31} & A_{32} & A_{33} \end{array}\right]\times \left(\left[\begin{array}{c} raw\;A_{x}\\ raw\;A_{y}\\ raw\;A_{z} \end{array}\right]-\left[\begin{array}{c} B_{x}\\ B_{y}\\ B_{z} \end{array}\right]\right)$$

where $A_{x}$, $A_{y},$ and $A_{z}$ are the calibrated data. The first term of the equation is the transformation matrix; $raw\;A_{x}$, $raw\;A_{y}$, and $raw\;A_{z}$ are the raw accelerometric data; and $B_{x}$, $B_{y},$ and $B_{z}$ are the bias calculated for each axis, respectively. Values of the transformation matrix and bias terms were calculated using Magneto 1.2 software [30]. This method is widely used also for compensating other physical variables measured by MEMS IMU onboard sensors, such as magnetometers. For completing the calibration, a set of raw data was acquired placing the sensor in multiple random positions in space covering approximately a sphere. The virtual sphere should have a radius equal to the magnitude of the physical reference used. In our case, the Earth’s acceleration gravity vector equals 1 g. If the raw data are plotted in 3D graphs, the shape defined by datapoints is ellipsoid and not a sphere. This because the bias related to sensor offset distorts the ideal sphere.

### 2.3. Field Test

**Figure 3:**
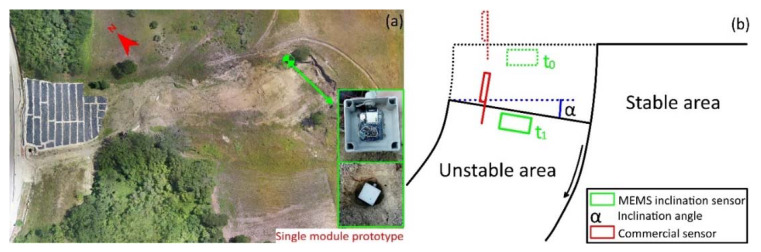
The (**a**) Pietrafitta landslide as seen from an orthoimage. The green point indicates the position of installation of the single-module prototype during the field test; Schematic (**b**) showing the geometry of deformational structures monitored and the expected kinematic of the movement.

After sensor optimization and prototype assemblage, a field test was conducted to evaluate system capabilities in a real landslide monitoring scenario. In this perspective, the system was installed at the upper part of the Pietrafitta landslide in the Apennine mountains of Southern Italy [10]. This landslide is actively moving, showing extensional features that form as a consequence of landslide material stretching [7] (Figure 3). In these conditions, landslide material deformation occurs with the formation of fault-like discontinuities accommodating spatial variation in landslide velocity [6]. At the landslide surface, fault dislocation is consistently related to depletion and rotation along an axis parallel to the fault plane (Schematic of Figure 3). The field experiment has been designed to measure such a rotation. For this purpose, the *x* axis of the sensor has been aligned to this virtual rotation axis.

The field test began on 20 September 2018 and had a total duration of 43 days. In this period, the results of inclination and its direction were recorded every 30 min. For validation purposes, a commercial tilt sensor was installed next to the prototype. This sensor, a 4–20 mA STRAGO modular inclinometer sensor [31], was connected to a dedicated logging system and powered by a battery and solar panel. The single-module inclination-monitoring prototype was installed within a hole, about 40 cm deep, excavated in the area chosen for the test (Figure 3). After prototype positioning, the hole was filled with a polyurethane spray foam that allowed to block the monitoring system in the ground whilst ensuring an easy removal after the end of the experiment. The commercial system was installed on a metal rod driven into the ground to a depth of 1 m. The *x* axis of the commercial sensor was aligned with the virtual axis of rotation of the deformational structures.

**Figure 4:**
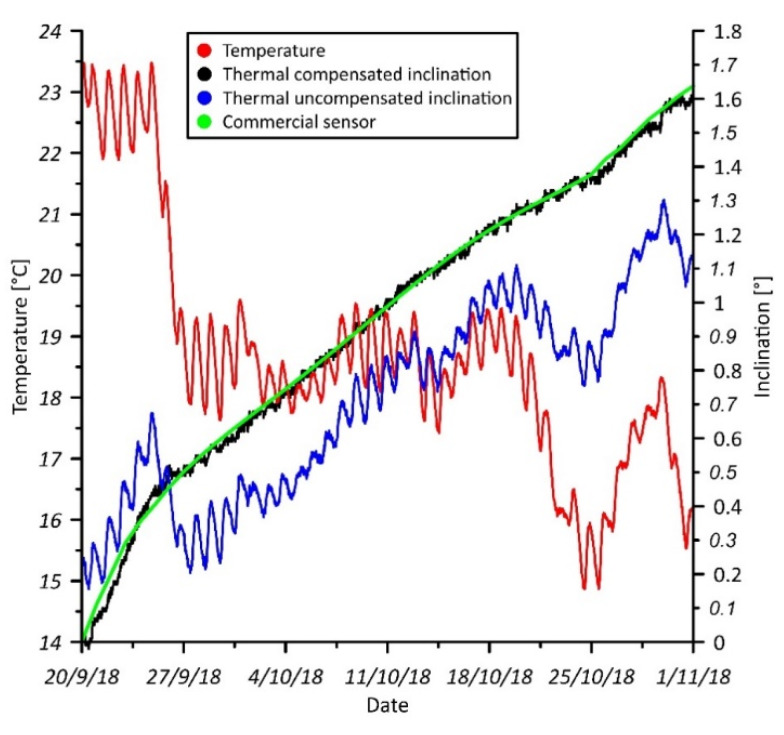
Graph showing monitoring data registered by the single-module prototype and the commercial sensor. The red, blue, and black lines represent the data registered by the single-module prototype, while the green line represents the inclination measured by the commercial sensor.

Results from field test are reported in Figure 4. The red line represents the temperature data registered by the onboard temperature sensor of the MEMS IMU. The blue and black lines represent the uncompensated and compensated inclination data registered by the prototype, respectively. The green line reports also the inclination values registered by the commercial sensor. The uncompensated inclination curve has a strong variability related to temperature trend, and the time series is completely different in comparison with that registered by the commercial sensor. Conversely, the compensated data show a good agreement with that registered by the commercial sensor indicating the efficiency of sensor optimization in removing drifting caused by temperature fluctuations. Especially, the evaluation of the RMSE indicated a final error estimated in 0.02°. Such low error indicates the potential of MEMS accelerometer sensors to be use in tilt-based monitoring applications, where medium to high accuracy is required.

## 3. Multi-Module Inclinometer

### 3.1. System Structure

The in-place inclinometer is composed of three parts: a chain of modules, each containing a measuring unit composed of a MEMS IMU sensor array and a low-cost Arduino^®^ Board; a master board responsible for acquiring, elaborating, and storing data measured by each single measuring unit; and a power system. Especially, since landslides are often located in remote places, the power system is composed of (i) a 50 W solar panel; (ii) a 12 A lead battery for storage energy; (iii) a charge controller; and (iv) a DC–DC voltage converter to adapt the 12 V of the battery into 5 V like Arduino^®^ alimentation. With this kind of configuration of the power system, the in-place inclinometer is suitable also for long-term observation. As developed, the overall cost of a single module is around €100, the cost of the master station is approximately €120, and the cost of the power supply is around €80. On this basis, an inclinometer composed of five modules, able to work in a landslide that is 5–10 m deep, has a cost of approximately €700. It is important to notice that the reduced cost of the developed system is only related to the system itself. Indeed, the installation costs are essentially equal to those of a commercial system.

The system, in its design configuration, has a total of five modules (i.e., measuring units) that forms the measuring chain. Measuring units are connected to each other with both a power and data transfer cabling system and a steel cable that, as a function of its length, permits to modulate the total monitoring depth of the chain and measuring resolution. The cabling system, connecting the master station with the measuring unit chain, is formed by four single pole cables. Of these cables, two poles are used for the power supply, and the remaining two are used as a unique cable communication bus. This configuration allows a simple and efficient cabling between the master station and the measuring units. Each measuring unit is protected by a steel case, equipped with guides that make the installation and the correct orientation easier. The multi-module inclinometer requires a standard inclinometer tube for being installed. This is necessary to ensure consistent orientation of the modules. The use of a standard installation scheme ensures the practicability of the proposed system in all of the field situations in which an inclinometer-based system is preferred for landslide monitoring and allows the installation of the developed monitoring system in monitoring sites already set up with a borehole inclinometer tube.

### 3.2. Module Hardware and Software

**Figure 5:**
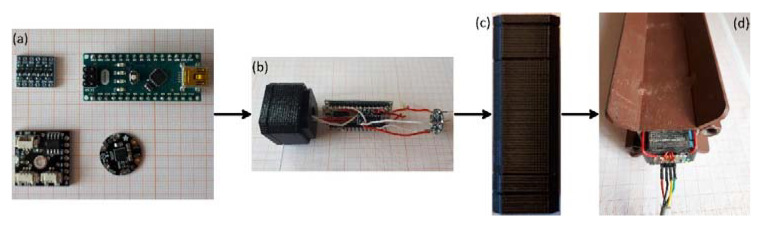
Diagram showing components and assemblage phases of the inclinometer modules. Especially, (**a**) shows the electronic components used; (**b**) shows the first assembly phase of the electronic components in the Acrylonitrile Butadiene Styrene (ABS) frame; (**c**) shows the appearance of the measuring unit composed by electronic components within the plastic housing; and (**d**) shows how a measuring unit is installed within the external steel case.

A module is composed of a measuring unit and an external steel casing. A measuring unit is composed of (i) an LSM9DS0 MEMS IMU; (ii) an Arduino^®^ NANO board [32]; and (iii) a communication interface (Figure 5). Each MEMS IMU was optimized following the procedure described for the single-module prototype in order to mitigate both the thermal drifting and the measuring offset (see above and [20]). The Arduino^®^ NANO board was selected for its dimension and reading capabilities and was programmed to read the data from the onboard accelerometers, apply a specific filter, and send them upon request to the master station. For data reading, the digital I2C communication protocol with a sampling rate of 100 Hz was used. Since the used MEMS IMU works at 3.3 V and the reading interface of the Arduino^®^ NANO board works at 5 V, a logic level shifter was used to ensure a correct voltage level communication.

**Figure 6:**
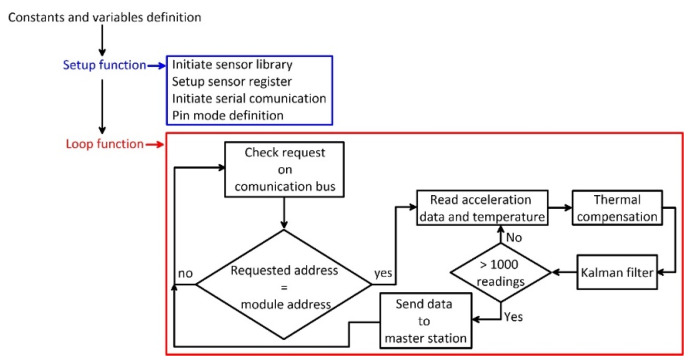
Flow chart showing the logic of the code of each measuring unit. In this case, due to the logic of data reading and acquisition, the loop is divided in two parts, the first for receiving the measure request by the master station and the second for reading sensors and sending data to the master station.

The logic of the code (reported in the Supplementary Materials) developed for making the module work is reported in Figure 6. Specifically, after first initialization of the variables and the libraries, the reading loop begins. The loop foresees a continuous reading of the measuring unit communication bus and is designed for reading data and sending them to the master station only under a specific address request. After the request of the master station, the Arduino^®^ NANO board (Arduino S.r.l., Monza (MB), Italy) reads the accelerometer raw data from the onboard IMU sensors. Such data are acquired and optimized for removing potential drifting. Low-degree polynomial equations, parameterized during the thermal analysis of each specific MEMS IMU, were used for this purpose following recommendations of authors of [20]. After thermal optimization of acquired data, compensated data are filtered using a single-stage Kalman filter implemented in the code as a filtering pre-processing stage. To ensure filtering performance, 1000 acceleration readings are used for each inclination measure. Data filtering is needed because the estimation of the orientation in the space of the MEMS IMU requires only the knowledge of the component of the static Earth’s gravity acceleration. That is why potential dynamic acceleration components deriving from external excitation sources (i.e., vehicular vibration, anthropic vibration, etc.) have to be identified and removed. The Kalman filter is particularly suitable for this purpose being widely used for removing noise in low to high frequency data for many applications [33,34,35,36,37,38]. In this case, Kalman filter parameterization has been completed using a trial-and-error procedure driven by standard deviation estimation. In particular, a Q parameter of 1 × 10^−3^ and an R parameter of 7000 were used. Once compensated and filtered, data are sent by the Arduino^®^ NANO to the master stations. The use of a data-upon-request strategy is crucial for ensuring the correctness of the displacement estimation and to make the system able to work in real conditions, especially considering the number of issues that need to be solved to avoid data loss. In this perspective, an important problem is related to the maximum distance of correct transmission of electrical pulses between two boards (i.e., the measuring unit and the master board).

**Figure 7:**
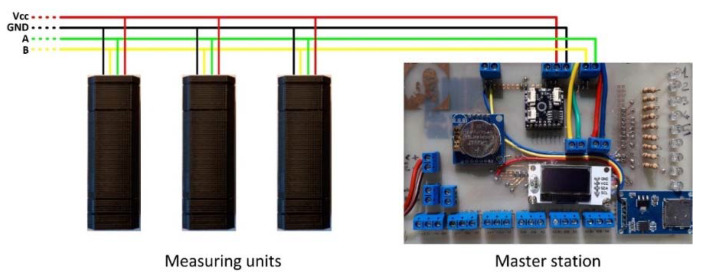
Wiring scheme for connecting the measuring units and the master station.

Despite the low-cost nature of the hardware used for the development of the inclinometer, the Arduino^®^ boards integrate different communication interfaces, such as the I2C and SPI protocols. Such protocols, although widely used for sensor communication, have the common limitation of transmitting data over low distances. Thus, for the development of the multi-module system, a specific hardware-based communication, characterized by greater stability over long distances was used. The selected interface is the RS-485 communication interface (Futura Elettronica s.r.l., Gallarate (VA), Italy) [39], supported by external hardware connected to the Arduino^®^ in a master/slave addressable setting. The RS-485 standard allows a communication distance up to 1200 m using only two wires, defined as A and B (Figure 7). A single communication line (two wires) supports up to 64 devices. Since RS-485 does not define a standard communication protocol, a software communication protocol allowing the transmission of inclination data from each inclinometer module to the master station, has been implemented. Especially, the communication mechanism is based on a protocol of “CALL” by the master station and “RESPONSE” by each inclinometer module. A limitation of the RS-485 communication is the limit of only two devices connected simultaneously. For this reason, the master has to make successive “CALLs”, one for each module until all of the modules are read in sequence.

Each measuring unit is housed within a specifically designed plastic frame that is guarded by an external steel frame. The plastic frame has been realized by 3D printing and is formed of multiple frame components. Each component is specifically designed to house a different electronic device (i.e., the sensor, the Arduino^®^ Nano and the RS-485 board). Each frame part was designed so that it can be assembled together with the others to form a single measuring unit. The material used for plastic frame printing was ABS plastic. Since measuring units forming the chain of the inclinometer are typically installed below the ground surface for common landslide monitoring application, and a measuring reference direction needs to be selected, an external, orientable steel cover was built for protecting and orienting the internal plastic frame. The steel cover was realized with steel, squared-section, tubes. The ensure waterproofing, epoxy resin was injected within the frame to fill the empty spaces. The measuring unit mounted within the steel casing forms the module. Figure 5 shows the different phases of module mounting and the final result.

As designed, the length of the single module including its protecting steel case was equal to 20 cm. For physical connection between successive modules, a stainless-steel eyelet terminal has been soldered at the center of the upper and lower covers of each steel case, so that a steel cable with a diameter of 3/5 mm can be used. The use of a steel cable to connect each individual module to form an inclinometer chain allows the modulation of the distance between the modules, so that the system offers some flexibility in adaptation to site conditions (e.g., the spatial resolution required, the depth of the sliding surface, etc.). For orientation and considering the common geometry of inclinometers tube, the external steel frame was prepared with four metal guides. This allows a correct installation and orientation inside a standard inclinometer tube.

### 3.3. Master Station Hardware and Software

All of the modules that form the inclinometer chain were connected to a master station.

The master station was formed by an Arduino^®^ Mega (Arduino S.r.l., Monza (MB), Italy) [40] and a self-made specific interface board circuit (PCB) (the schematic circuit is reported as Supplementary Materials) installed on the pinout of the Arduino^®^ Mega. This station has the function of acquiring acceleration values measured by axial accelerometers of the MEMS IMU, installed within each module and read by the Arduino^®^ NANO board thought the RS-485 interface and communication protocol. In addition, the interface was designed also for connecting and reading additional sensors. Indeed, both analogue and digital sensors like water content probe, pore water pressure transducer, temperature probe, and rain gauge can be associated to the inclinometer chain, in order to measure additional environmental variables that might drive landslide movement, allowing correlation to slope deformation rate.

**Figure 8:**
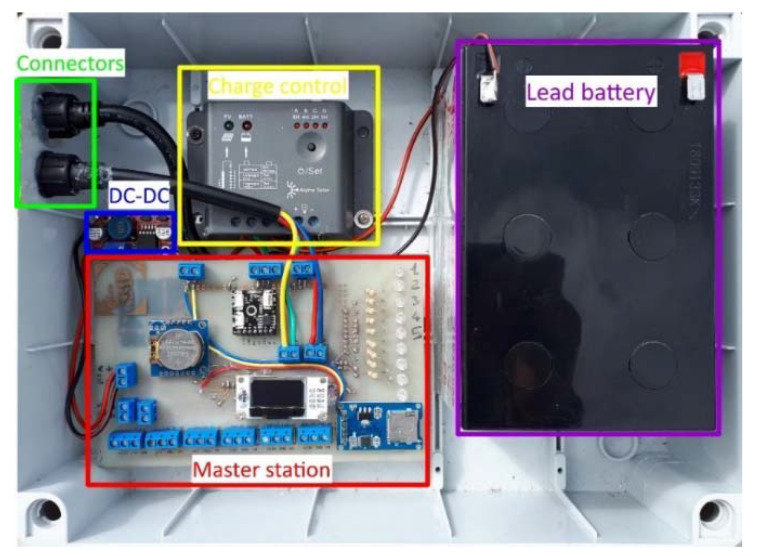
Master station and power supply mounted within the waterproof box. Waterproof connectors for measuring unit and solar panel connection are visible.

The developed board interface is composed of (i) a communication RS-485 interface, the same as that installed within each module; (ii) an Real Time Clock (RTC) clock connected via I2C bus; (iii) a micro SD module that is the local storage of the data; (iv) a 0.96 inch Organic Light Emitting Diode (OLED) display that allows displaying the main information about the status of the health of the modules and the registered deformation data (i.e., tilt, direction, and displacement), (v) nine LEDs programmed as a modules status check; and (vi) a number of connectors for interfacing other sensors or additional Arduino boards (e.g., for remote communication features). The master station is mounted within a waterproof box in association with power supply devices (Figure 8).

**Figure 9:**
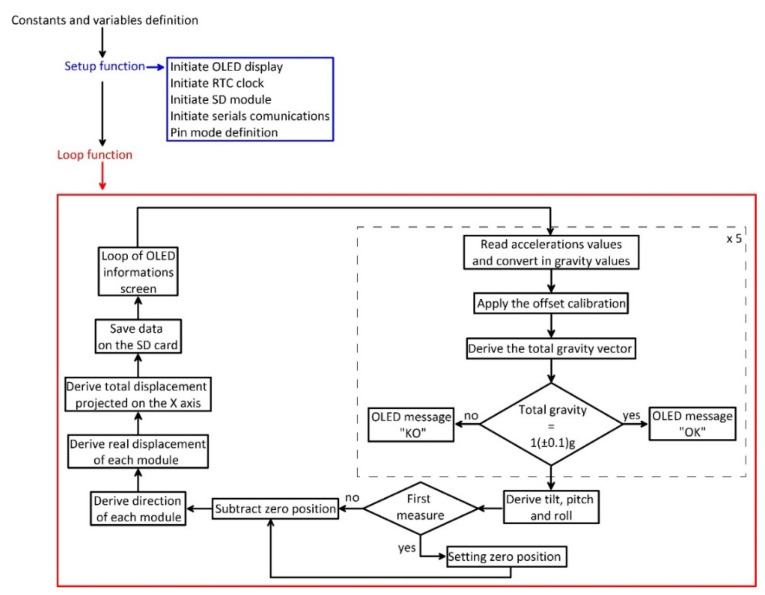
Flow chart showing the logic of the code of the master station.

The logic of the code (reported in the Supplementary Materials) developed for operating the master station is reported in Figure 9. After first initialization of the variables and the libraries to activate the interfaced boards (i.e., OLED display, SD card adapter, RTC clock, and communication device), the loop begins. The master station first requests data from the modules through a specific request on the communication bus. Each request provides readings from a single measuring unit characterized by a specific addressed. Received raw acceleration data, already compensated for thermal drifting and filtered for dynamic acceleration components, are converted in gravity values, and the offset is removed. At this point, the code is designed to check for the correctness of the gravity data through Equation (4). In case of an error, the corresponding led on the master station turns red, and the error is indicated to the user on the OLED display.

After acquiring data from each module, the station is programmed for converting such data in inclination values using Equations (1)–(3), implemented in its onboard software. In particular, these equations derive inclination angles along the three axes from acceleration data for each sensor. Subsequently, inclination data are converted in displacement at a specific depth d (i.e., relative displacement to a specific depth/module) using the equation below:

(6) d=L×sinθ

**Figure 10:**
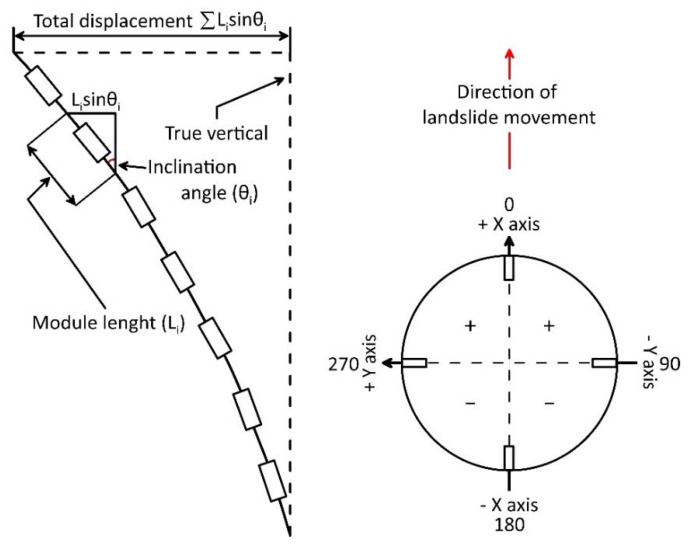
Calculation scheme for a multi-module inclinometer (modified from [41]).

In this equation, L is the length of the module, and θ is the inclination of the module in comparison with the Z axis. The length of the module corresponds to the distance between two successive modules, and the reference segment for displacement estimation is reported in Figure 10. On this basis, the L parameter can be derived using the following equation:

(7) $$L=L_{m}+L_{c}$$

where $L_{m}$ is the length of the external steel case of the module, and $L_{c}$ is the length of the steel cable used for connecting two successive modules.

Since displacement at different depths is estimated as the difference in inclination between two successive measurements, the first set of data is used to set the reference position. Subsequent measurements are used to compute the relative displacement in comparison with the first measurement. For each reading, the master station computes displacement and its direction for each module. Such data are then used for calculating the cumulative displacement along the chain (i.e., resultant). In Figure 10, the scheme of an inclinometer column and the ground surface displacement derived value are shown. The total displacement at the head of the multi-module inclinometer is equal to:

(8) $$\sum L_{i}\times sin\theta_{i}$$

where $L_{i}$ and $sin\theta_{i}$ are the module length (that is commonly constant along the chain) and the inclination angle derived by acceleration measurements completed by each measuring unit, respectively. On this basis, a total displacement value at the head of the inclinometer chain and data for constructing relative and cumulative displacement curves are provided. These data are saved on the local memory storage (i.e., an SD card), and a print loop is started providing the tilt measured data values for each module on the OLED display.

## 4. Laboratory Testing

After inclinometer assemblage, a test in controlled conditions of the developed instrumentation, oriented to validate sensors’ linearity and offset correction, thermal efficiency, and general measurement accuracy was completed.

### 4.1. Linearity and Offset Tests

**Figure 11:**
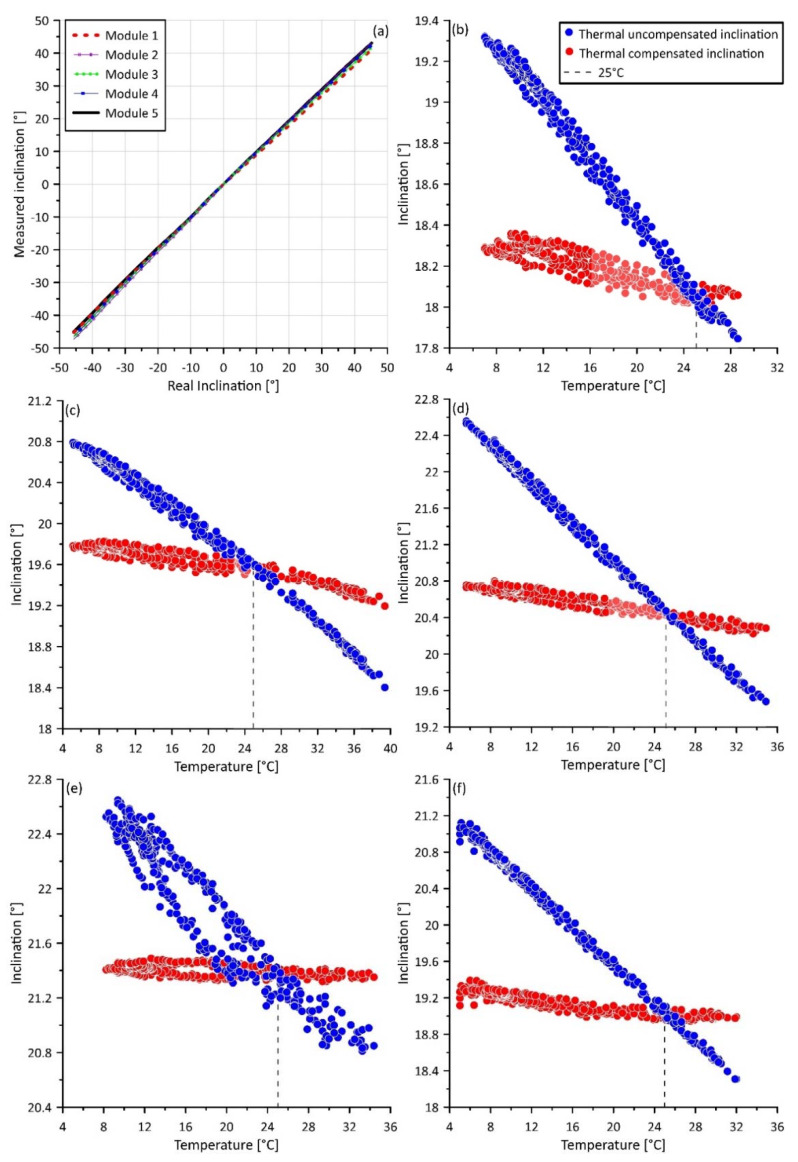
Graphs showing results of the thermal efficiency, linearity, and offset tests. Graph (**a**) shows results of linearity and offset tests. Graphs (**b**–**f**) present data derived by the thermal efficiency test of modules 1, 2, 3, 4, and 5, respectively.

As the first step in system testing, the linearity and efficiency of offset correction were tested for each module (i.e., each equipping sensor) in an established range of variation of the inclination. This procedure was completed through a precision inclined plane. Such a plane allows to set inclination with an accuracy of ±0.25°. Especially, we completed multiple inclination measurements in the range of −45 to 45° with an inclination step of 5°. The result of this test shows the absence of non-linear deviation in the measurement of tilt and an absence of an offset (Figure 11a). The absence of the offset is an evidence of the good efficiency of the parameters utilized in Equation (5) derived from the calibration of the MEMS IMU onboard sensors.

Results of the test also indicate the linearity of the measuring system. This linearity is expected also as a consequence of the calculation scheme used for deriving tilt information from accelerometric measurement (i.e., Equations (1)–(3)). This scheme, considering the acceleration measured along all of the three axes to derive inclination, allows for an improved sensitivity and accuracy of calculation [27,42]. This is also related to the use of the arc tangent operator [26] (i.e., 1, 2, and 3). In addition, data derived by inclined plane test were used to estimate the measuring error in the form of RMSE. Specifically, the RMSE was calculated for three angular ranges. The first was between −10 and 10°, the second was between −20 to 20°, and the third is between −45 to 45°. Table 1 reports the RMSE estimated for each measuring unit. The lower values of RMSE (i.e., higher accuracy) correspond to a lower angular range. This behavior is expected from low-cost MEMS IMU onboard accelerometers used for measuring inclination. However, considering that field monitoring applications, particularly landslide displacement tracking, require the measurement of inclination values typically in the range of ±20°, this kind of system can be considered suitable for continuous monitoring in real field conditions.

### 4.2. Thermal Efficiency Test

As a second step of system testing, the thermal efficiency of the system was tested considering a natural temperature variation between 5 and 40 °C. For this purpose, the modules were exposed, in external environmental conditions, to natural, uncontrolled temperature variation for a total duration of 72 h. The modules were kept in a fixed position so that the measured variation in inclination was potentially related to temperature variation. Inclination data were recorded every 10 min.

Figure 11b–f reports results from the uncontrolled thermal efficiency test. Specifically, the graphs show both the thermally compensated and uncompensated inclination data, measured by each measuring unit (red and blue dots, respectively). Since sensors are calibrated by the manufacturer to work at 25 °C, the compensated and uncompensated datasets have the same tilt value at this temperature. The observation of this effect can be considered an indication of the correctness of the measurements. Both datasets were used to derive an estimation of the RMSE error in comparison with the real values measured at 25 °C and its reduction after sensor optimization through the application of the thermal compensation. The RMSE and its variation is presented in Table 2. The reduction of the RMSE due to sensor optimization in a thermal drifting perspective is consistently high, varying between 74 and 86%. In addition, Figure 11b–f provides an overview of how the application of a thermal compensation strategy in sensor optimization can reduce the thermal drifting of low-cost MEMS accelerometric sensors, making them suitable for operational monitoring in natural field conditions.

It is important to notice that there are a number of MEMS sensors that, being developed for this scope, can be used in environmental monitoring applications without particular optimization [43,44]. However, these high-performance sensors, characterized also by a high accuracy and stability, have a very high cost and in some case can suffer additional drawbacks related to the reading interface and cabling system. Moreover, the electronic interface can be very complex and poorly customizable. Conversely, the Arduino^®^ board is highly flexible allowing development of specific code and integration of a multitude of libraries that provide support for solving a number of problems of variable complexity.

### 4.3. Multi-Module Inclinometer Test

After testing the measuring units for linearity, offset, and thermal efficiency under natural temperature variation, the inclinometer chain in its final configuration was tested for evaluating its measurement capabilities. This test, conducted in an indoor limited temperature variation environment, allowed to evaluate the global behavior of the system and to show expected measurement results and associated accuracy in a continuous monitoring landslide scenario. For this purpose, a supporting frame allowing differential alignments of inclinometer modules was used. It was composed of a 118 cm × 200 cm wood panel frame, specifically designed to allow a controlled displacement at different distances from its base of the inclinometer column and regulation of its shape. For testing, each module was kept fixed at different positions through the installation of specific struts. On this basis, the test permitted to obtain a number of different synthetic deformation curves that form a basis for checking the accuracy of the instrumentation in measuring relative and cumulative displacement along the chain. An evaluation of measurement accuracy was completed through a comparison between the inclination measured by each module and its real position in the space. During this test, measurement repeatability was analyzed. Especially, reference inclination of each module was derived as the arithmetic average of 5 distinct measurements completed for each fixed position. Such measurements were used to derive the standard deviation.

**Figure 12:**
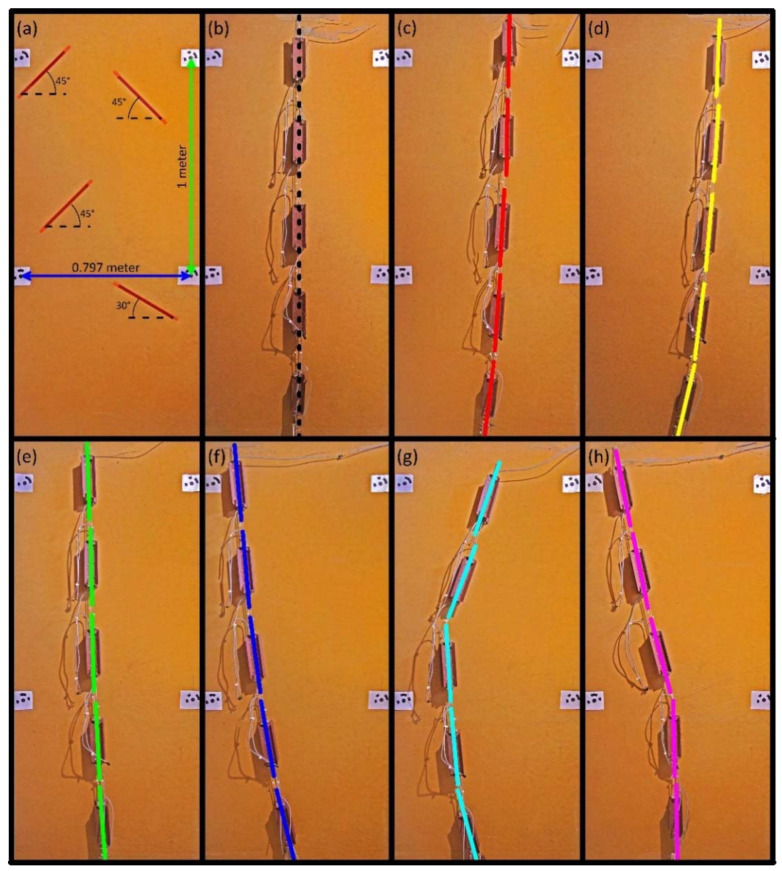
Configurations and results of measurement tests of the multi-module inclinometer. The multicolor segments represent inclination registered by each module in each simulation. (**a**) Linear markers used for the validation of the method of analysis; (**b**–**h**) synthetic curve derived by the analysis of orthoimages in GIS environment representing the shape in operative conditions of the multi-module system; The geometric configuration of (**b**) is the reference for measuring displacement simulated by synthetic configurations (**c**–**h**).

For this purpose, after defining the synthetic geometry of the chain, a high resolution orthoimage (300 dpi) was developed on the basis of multiple ground-based photos. The objective of this procedure was to obtain a high-accuracy model of the system and reproduce its spatial arrangement allowing to measure module inclination in a GIS environment. This method was selected because of the orthophoto’s capability of being a good base for orthographic measurements, due to their intrinsic equivalent dimensional ratio. Orthoimages were derived from multiple sets of three pictures taken from the same distance from the wood panel, using a reflex camera with 16 MP resolution (Pentax K5) and an aspherical lens characterized by a focal length of 17 mm. Such pictures were converted into orthoimages using the Agisoft Photoscan Professional [45] environment and considering four targets of known cartesian coordinates (Figure 12a). Before using the obtained orthoimages for deriving module inclination, the method was validated for its accuracy comparing the known position and inclination of a number of linear markers reported on the supporting wood panel with their position and inclination derived by the orthoimages. Figure 12a reports the result of the validation test based on distance and angular measurements completed considering the positions of such markers. This test indicates a very low error in measuring inclination of linear markers and their length (i.e., ±0.345° and ±0.3 cm) also indicated by the overlap of red segments on the black lines. The good/optimal overlapping between the linear black marker and red segment underlines the validity of our method of analysis.

Figure 12b–h shows the synthetic configurations of the system for the test. Especially, seven different configurations were used and corresponding orthoimages were developed. The test reproduced a range of inclinometer deformation curves that can occur in real cases of landslide monitoring applications. For instance, Figure 12h reproduces the most common deformation curve that can be expected in a translational landslide in which the system is installed within a borehole reaching a depth higher than that of the basal slip surface. In this condition, the deformation of the upper part of the chain and the substantial stability of deeper modules is expected. Table 3 reports results of the test in terms of module inclination as measured by the system and derived by orthoimage analysis into a GIS environment. As indicated in the table, the estimated RMSE ranges between ±0.162 and ±0.304° indicate an estimated displacement measurement accuracy of ±0.37%/m. Considering that the test was conducted simulating different typical landslide deformation patterns, we expect that the estimated accuracy is representative of system performance also in real landslide scenario applications. During this test, the measurement repeatability of the system was analyzed. For each module and configuration, five measurements were completed. A total of 175 measurements were aggregated to calculate the average standard deviation of the system. It was estimated to be ±0.012°.

**Figure 13:**
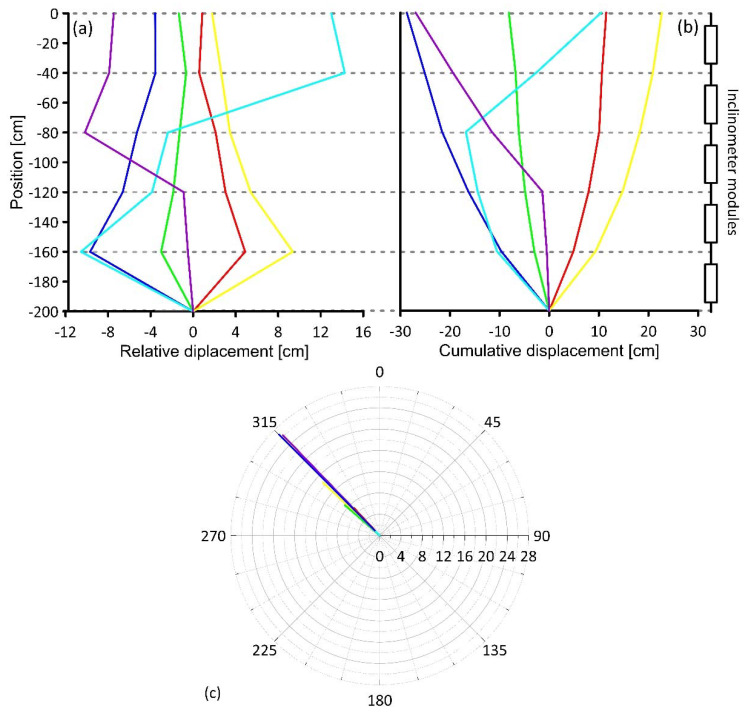
Graphs showing results from inclinometer measurements reproduced in the test. (**a**) Results in terms of relative displacement; (**b**) results in terms of cumulative displacement; polar graph (**c**) depicts the direction of cumulative displacement from the base to the head of the column.

Figure 13 shows the final results provided by the system, developed following standard indication ASTM D6230-13 [46], in the form of relative and cumulative displacement and displacement orientation. Especially, Figure 13a reports the relative displacement along the chain, measured by each module. Figure 13b reports the cumulative displacement along the chain calculated considering the displacement derived by inclination measurement completed by each module. In the polar graph of Figure 13c, the direction of the cumulative displacement is plotted from the base to the head of column. It is important to notice that, the relative displacement representation allows to identify the location of a potential deformation or shear zone [47].

## 5. Conclusions

In this paper, a new, low-cost, and open-source multi-module inclinometer system for continuous landslide monitoring is presented and tested. The system is composed of multiple modules, formed by a measuring unit, a protecting steel case, a specifically developed master station, and a power system. Each module, responsible for measuring inclination at different depths within a borehole, is formed of an LSM9DS0 MEMS IMU, an Arduino^®^ NANO, and an RS 485 interfacing board. The selected MEMS IMU is equipped with multiple axial accelerometers that, after thermal and offset optimization, can be used to measure components of Earth’s gravity and derive module inclination and inclination direction. The master station developed, on the basis of an Arduino^®^ MEGA, for acquiring data from each module under specific address request is responsible for converting optimized acceleration data into inclination, calculating relative and cumulative displacement, and deriving displacement direction. The power system is developed for providing continuous supply in order to make the system able to support continuous monitoring capabilities also in remote locations.

System testing was conducted in controlled conditions through a specific and reproducible strategy accounting for linearity and offset, thermal efficiency, and displacement measuring error estimation. It indicates high performance with an inclination RMS error ranging between ±0.162 and ±0.304° and a final displacement measuring accuracy of 0.37% of the length of the inclinometer chain. The linearity test indicates the high linearity of the measures, especially in the range of ±20° that is the typical operating range of such kind of instrumentations. The thermal efficiency test indicates the high efficiency of the system in preventing measuring errors caused by thermal drifting. A forthcoming paper will describe results from a field test conducted within a set of actively moving landslides and discuss advantages and drawbacks of the multi-module system in real field conditions.

## Figures and Tables

**Table 1:** RMSE values estimate for each module through precision inclined plane test.

RMSE (±°)	Module 1	Module 2	Module 3	Module 4	Module 5
±10 °	0.22	0.16	0.20	0.16	0.12
±20 °	0.61	0.47	0.55	0.45	0.35
±45 °	1.54	1.19	1.42	1.13	0.89

**Table 2:** RMSE calculated on the basis of data acquired in natural, uncontrolled temperature variation conditions in the range between 5 and 40 °C. The table provides both RMSE derived by thermally compensated and uncompensated measurements and variation of the RMSE after compensation.

RMSE (°)	Module 1	Module 2	Module 3	Module 4	Module 5
Thermal compensated	0.17	0.18	0.19	0.18	0.10
Thermal uncompensated	1.33	0.83	0.84	1.28	0.73
Variation (%)	87	74	77	86	86

**Table 3:** Inclination data measured by each module in different geometric configurations of the inclinometer chain.

Image	Module 1		Module 2		Module 3		Module 4		Module 5	
	Est.	Meas.	Est.	Meas.	Est.	Meas.	Est.	Meas.	Est.	Meas.
(c)	1.40	1.23	0.94	0.81	2.98	3.04	4.00	4.37	7.09	7.03
(d)	2.41	2.56	3.76	3.79	4.83	4.97	7.71	7.78	13.31	13.56
(e)	−1.89	−1.92	−0.85	−0.93	−1.97	−1.81	−2.72	−2.66	−4.16	−4.31
(f)	−5.44	−5.13	−4.98	−5.08	−7.76	−7.58	−9.64	−9.95	−13.72	−14.02
(g)	19.27	18.95	20.75	20.89	−3.28	−3.39	−5.36	−5.54	−15.48	−15.30
(h)	−10.56	−10.76	−12.09	−11.38	−15.01	−14.75	−1.07	−1.27	−0.82	−0.70
RMSE (°)	±0.22		±0.30		±0.16		±0.21		±0.19	
Accuracy	±0.38%/m		±0.53%/m		±0.28%/m		±0.36%/m		±0.33%/m	

## Supplementary Materials

The codes developed for making both the modules, and the master station working, as well as the schematic of the master station are available online at https://www.mdpi.com/1424-8220/20/11/3318/s1.
