# Supplementary figures and images for: A Multi-Module Fixed Inclinometer for Continuous Monitoring of Landslides: Design, Development, and Laboratory Testing
Source: Sensors (Basel). 2020 Jun 10;20(11):3318. doi: 10.3390/s20113318 (PMC7308859; doi:10.3390/s20113318)

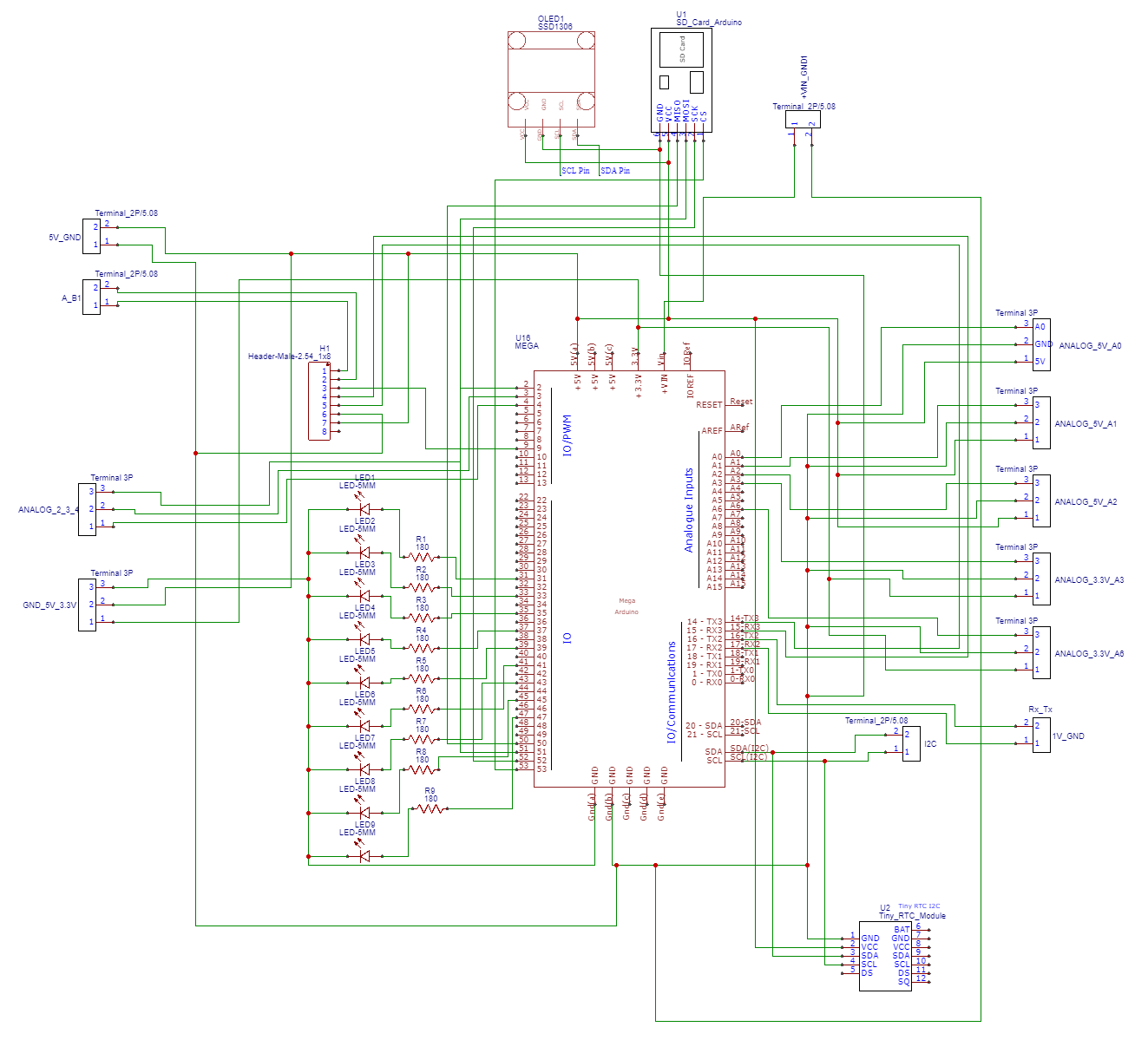

Supplement: Supplementary file 1 [file sensors-20-03318-s001.zip › sensors-825735-supplementary-v3/Supplementary/Master_Station/Schematic_Master_Station_Interface_Board.png]
